# A Combined Diagnosis and Treatment Algorithm for Spine Infection Management: A Single-Center Experience

**DOI:** 10.7759/cureus.28251

**Published:** 2022-08-22

**Authors:** Ahmet T Başak, Nazlı Çakıcı, Muhammet Arif Özbek, Mehdi Hekimoğlu, Önder Çerezci, Ozkan Ates, Tunc Oktenoglu, Mehdi Sasani, Ali Fahir Özer

**Affiliations:** 1 Neurosurgery, Amerikan Hastanesi, Istanbul, TUR; 2 Neurosurgery, Medicana Hospital, Istanbul, TUR; 3 Neurosurgery, Medipol University, Istanbul, TUR; 4 Physical Treatment and Rehabilitation, Amerikan Hastanesi, Istanbul, TUR; 5 Neurosurgery, Koç University School of Medicine, Istanbul, TUR

**Keywords:** lumbar spine, spine infection, modified united algorithm, intermittent irrigation, early stabilization

## Abstract

Background and objective

Spinal infection (SI) is an infectious disease affecting the vertebral column, spinal cord, and adjacent structures. The infection can occur following interventions or spontaneously. The aim of this study was to highlight the importance of employing a methodological approach for the accurate and rapid diagnosis of SI and to share information on the most effective treatment method, which involves using a diagnostic-treatment algorithm that can help with SI management.

Methodology

This study included 50 patients diagnosed with SI between 2016 and 2020. The treatment follow-up period was limited to six months, and the study was conducted as a retrospective cohort analysis. The sample consisted of 22 female patients and 28 male patients, and the mean age of the patients was 50.2 years. All patients received diagnosis and treatment according to the algorithm described in this article.

Results

In the study group, 60% of patients had an infection in the lumbar spine, 4% in the thoracal spine, 12% in the cervical spine, and 8% in the sacral spine. Previously operated patients were diagnosed on the 30.16th day on average. A total of 19 patients (38%) had no history of undergoing surgery. Radiologically, the most common finding was spondylodiscitis/discitis (32%). Osteomyelitis was detected in one (2%) patient. Methicillin-sensitive *Staphylococcus aureus* (MSSA) was the most commonly isolated organism in culture results and was detected in 13 patients (26%). The culture results of 12 patients (24%) were negative. The number of patients with active SI who were unstable and stabilized at the time of diagnosis was 11 (22%), and stabilization materials were removed in two patients (4%). In the 6th month of control, the patients did not have any complaints, signs of an infection, or unstable vertebral column.

Conclusions

We conclude that the combined algorithm we recommend for the diagnosis and treatment of patients with SI can prevent negative deviation and is an effective treatment for this condition.

## Introduction

Context

Spinal infection (SI) affects the vertebral body, intervertebral disc, and/or adjacent paraspinal tissues [1]. SI can develop as a result of trauma, spontaneously, or after surgery. There are typically three spreading pathways: hematogenic, direct contact to the outer surface, and spread from adjacent tissues. The increased incidence of SI may be associated with the development of imaging techniques and clinical diagnostic methods, increased number of immunodeficient patients, increased intravenous drug abuse, increased number of spinal surgeries, and instrumentations [2]. Due to its nonspecific presentation, early detection of SI is still difficult, and there are no known reports of a common treatment algorithm.

Epidemiology

The spine is prone to infection, and SI accounts for 2-7% of all musculoskeletal infections. The estimated mortality rate associated with SI is between 2 and 4% [3]. Previous spinal surgery, another infectious focus, diabetes mellitus, advanced age, intravenous drug use, HIV infection, immunosuppression, oncological history, renal failure, rheumatological diseases, and liver cirrhosis are the most common predisposing factors [4]. In recent years, the incidence of SI has been on the rise despite advancements in imaging and laboratory techniques.

Etiology

SI is divided into pyogenic or granulomatous etiologies and is caused by three primary agents: bacteria that cause pyogenic infection, tuberculosis or fungi that cause granulomatous infection, and, less frequently, parasite-induced infection. In the past, tuberculosis was the leading cause of SI, but its incidence has decreased over the past 50 years due to enormous success in the diagnosis and treatment of pulmonary tuberculosis. Today, the most common isolated pathogen is *Staphylococcus aureus*, with an incidence rate between 30 and 80% [5,6]. In some reports, Gram-negative bacteria such as *Escherichia coli* were found to be responsible for 25% of SIs [7]. *Mycobacterium tuberculosis (M. tuberculosis)* is especially common in patients with HIV and accounts for 60% of the pathogens identified in this patient group. Anaerobic agents also cause infection, especially in cases of penetrating spinal trauma. However, despite all efforts to identify the infectious agents, no pathogens have been identified in one-third of patients with SI [8,9]. In the Middle East, Eastern Europe, and the Mediterranean countries, the incidence of *Brucella* has increased significantly [10].

Pathophysiology

Pyogenic SI can occur in two ways: by the hematogenic path and by direct inoculation during interventions in the spine [11]. The most common hematogenic sources of pyogenic SI are the genitourinary system (29%) and soft tissue infections (11%). Surgical procedures such as lumbar puncture, facet injection, laminectomy, and discectomy can cause direct inoculation (15-40%). In rare cases, inoculation may occur from close surrounding tissues such as a retropharyngeal abscess or infected aortic graft (4%). *M. tuberculosis*, also known as Pott's abscess, is the most common factor in granulomatous SI. Compared with pyogenic SI, Pott's abscess tends to be more thoracically located.

The adult intervertebral disc is avascular, and therefore, a septic embolism can cause bone infarctions and the spread of infection to adjacent structures, resulting in the classic spondylodiscitis appearance. As a result of these changes, the risk of spinal instability, spinal deformity, and spinal cord compression increases [12]. The progress in infection can cause an uncontrolled spread to the surrounding tissues, leading to paravertebral and psoas abscesses. The spread of SI to the spinal canal can cause epidural abscesses, subdural abscesses, and meningitis. The spread of spondylodiscitis to posterior structures is rare due to the vascular anatomy of vertebrae and is usually seen in fungal and tuberculosis spondylodiscitis. The Hematogenic spread of pyogenic spondylodiscitis primarily affects the lumbar spine (58%), followed by the thoracic spine (30%) and the cervical spine (11%) [13]. The direct transmission pathway is often iatrogenic; with lumbar puncture, they are seen after invasive epidural intervention and surgery [14].

Diagnosis

Clinical

Diagnosis of SI is often difficult and requires considerable effort. Therefore, there is usually a significant delay between the emergence of initial symptoms and the establishment of a diagnosis. The diagnosis should be supported by clinical, laboratory, and imaging findings. Usually, the first clinical symptom is nonspecific back or neck pain, but 15% of patients may not experience this pain. Patients usually complain of pain that worsens at night, often associated with radicular pain in the chest or stomach. Of note, 48% of patients with pyogenic spondylodiscitis and 17% with tuberculosis spondylodiscitis tend to manifest fever [15], and infections in the cervical region can cause dysphagia and torticollis [15]. Approximately 33% of patients have neurological symptoms such as loss of strength in the extremities, numbness, and incontinence [16]. These symptoms are often associated with late diagnosis, cervical infection, presence of an epidural abscess, and tuberculosis infection [15]. During the physical examination, attention should be paid to kyphotic deformities and soft tissue swellings, which are often associated with tuberculosis spondylodiscitis [17]. Clinical presentation in the pediatric age group may be different and include the following symptoms: irritability; refusal to crawl, sit, or walk; abdominal pain; and incontinence [18]. Fever is a rare finding in children, and the most common finding is lumbar lordosis [18]. The development of neurological disorders is also rare in this age group.

Laboratory

In SI, the initial laboratory analysis generally shows leukocyte elevation in 42% of patients and erythrocyte sedimentation rate (ESR) elevation in 92% of patients. An increase in C-reactive protein (CRP) level is an acute-phase reactant in infection and inflammation. It rises within four to six hours, folds every eight hours, and peaks within 36-48 hours; because its half-life is 24-48 hours, it is effective in infection monitoring. However, because CRP level is an acute-phase reactant, it can rise in any inflammatory stage and reduce to its normal value within 5-10 days after surgery. ESR, like CRP, rises in response to inflammation and postoperative state; however, it takes 30-40 days to return to its normal value [19]. ESR is a highly sensitive but low-specific marker of infection. CRP also rises in more than 90% of patients with spondylodiscitis. The WBC count has low sensitivity and hence it is the least useful marker of infection [5]. When SI is suspected, it is recommended that blood and urine tests be performed before the administration of antibiotics [9]. While aerobic cultures are carried out routinely, tests of anaerobic cultures have not been routinely performed since the late 1980s because of the decreased incidence of anaerobic bacteria. As a result, centers today may not be equipped to study anaerobic cultures. However, anaerobic bacteria have re-emerged as a major pathogen, and it is highly recommended that appropriate tests are performed in response to the increasing positivity rates of blood cultures [20]. Results of blood cultures are positive in 58% of patients during the period of increased fever. Despite an obvious history, associated positive blood cultures, and imaging findings in line with the clinical diagnosis of SI, the definitive diagnosis can only be achieved by microscopic or bacteriological examination of infected tissues. It is particularly important in patients whose blood culture is negative or non-specific [5,6]. In patients with Pott's abscesses, purified protein derivative tests should be performed. The purpose of the purified protein derivative test is to reveal whether the patient has previously encountered tuberculosis bacillus. Because *M. tuberculosis* breeds slowly in cultures (six to eight weeks), blood plasma interferon-gamma level provides results in less than 24 hours and can be used for diagnosis.

Radiology

MRI is still the most reliable method for diagnosing spondylodiscitis because of its high sensitivity (96%), high specificity (94%), and ability to give detailed data on paraspinal tissues and the epidural cavity [21]. The most common area for pyogenic infection is the lumbar spine, followed by the thoracic, cervical, and sacral spine [8]. The most common area for tuberculosis infection is the thoracic spine [9]. The rate of infection in the spine is as follows: 48% lumbar, 35% thoracic, 6.5% cervical, and 5% thoracolumbar and lumbosacral. Whereas MRI imaging alone is as sensitive as radionuclide imaging (96%), it can also distinguish between malignancy and infection at a very high rate (93%). In Pott's disease and spondylodiscitis, endplate and disc involvement are at the forefront, whereas in malignancy, corpus involvement and spread to soft tissue are more common. Edema seen in fat T2 and short tau inversion recovery images is an early indication of infection. Loss of height and contrast involvement in the disc has a high sensitivity for infection (70-100%). The disadvantage of MRI is that it can give false-negative results for epidural abscess, as it cannot evaluate the entire skeletal system and the cerebrospinal fluid is isointense with abscess [22].

Plain X-rays, however, should be obtained in every basic assessment. Although X-rays have low specificity (59%) in the diagnosis of spondylodiscitis, they can be helpful in detecting irregularities of vertebral endplates and/or low intervertebral disc height in advanced cases. Flexion/extension X-rays can be useful in the follow-up period for detecting a possible instability. CT scan can detect early changes in the endplates and bone necrosis. However, the onset of symptoms of bone destruction, which causes a pronounced delay in diagnosis, can make CT a less effective diagnostic tool for SI [22]. CT scan is the most useful imaging technique for evaluating early changes of vertebral end plaques, the presence of bone necrosis, and pathological characteristics suggestive of tuberculosis [22]. Although MRI is the gold standard in SI diagnosis, there is no pathognomonic finding that can make a definite distinction between SI and possible neoplasm. In patients with suspected SI, complementary diagnostic methods can be applied [23], such as sequence bone/gallium imaging and the 67Ga-SPECT form radionuclide diagnostic method. Because of the low specificity of these two techniques, the use of fluoro-2desoxy-D-glucose positron emission tomography, which can be a promising technique for diagnosis, has increased since degenerative changes and fractures do not normally show fludeoxyglucose intake. However, today, the diagnosis of radionuclide should be separated only for patients with an indefinite diagnosis or special follow-up [23]. Tuberculosis spondylitis has a pattern in which the intervertebral disc is relatively protected and the vertebral body has heterogeneous involvement, a large paravertebral abscess, and major bone destruction. Radiological changes can occur later and consist of the loss of gadolinium intake and bone restoration, not always compatible with the resolution of infection. Therefore, despite the increased use of MRI for follow-up response to treatment in patients with SI, some studies do not recommend the routine use of MRI in follow-up. These studies report that although clinical treatment is improving, some MRI findings may persist or worsen during treatment and lead to unnecessary invasive treatments [24].

## Materials and methods

Evidence acquisition

**Figure 1:**
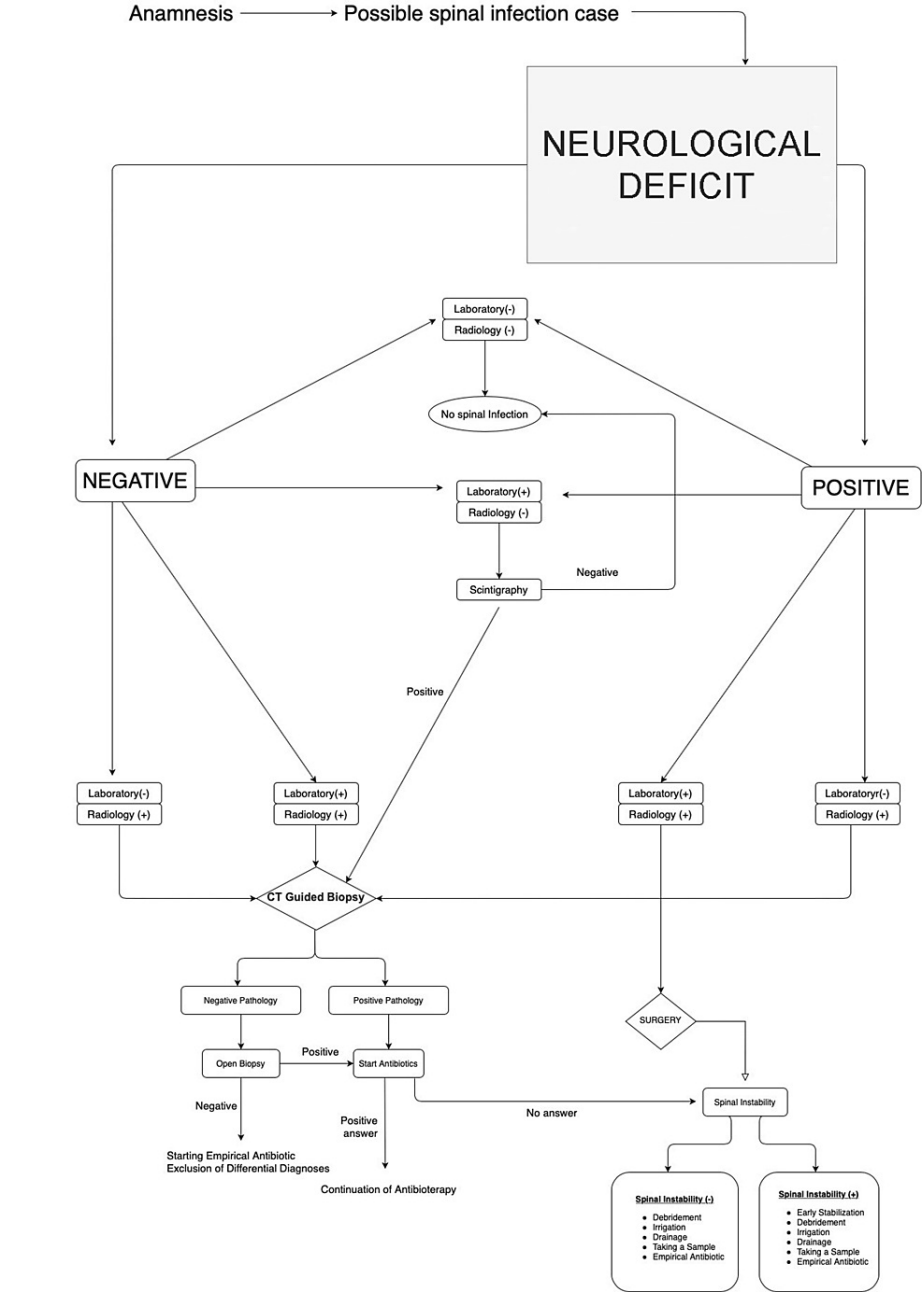
Diagnosis and treatment algorithm

Between 2016 and 2020, 50 patients diagnosed with SI at our clinic were included in this study. The duration of follow-up treatment was limited to six months, and our study was carried out retrospectively. The study included 22 female and 28 male patients, with an average age of 50.2 years. All patients in our study received their diagnosis and treatment based on a modified algorithm illustrated in Figure 1.

**Figure 2:**
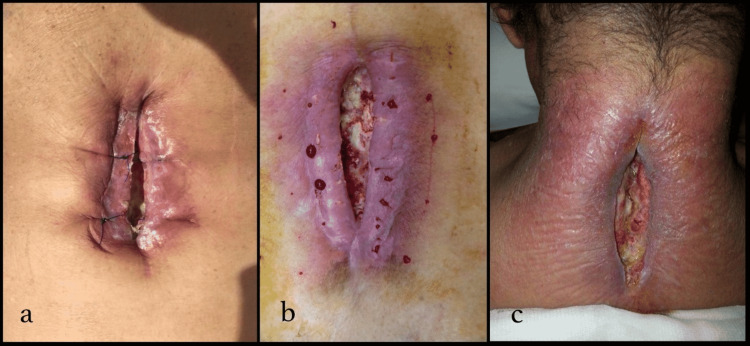
Wound site infections after various procedures a) Wound site infection after lumbar microdiscectomy. b) Wound site infection after lumbar microdiscectomy and keloid formation. c) Wound site infection after cervical laminectomy and posterior stabilization

In possible infection cases, the first point of importance is the presence of neurological deficits. If so, the patient undergoes surgery regardless of the presence of the stated infection. But if the neurological deficit is negative, the patient undergoes diagnostic procedures. Infection in isolation can be treated by antibiotics. However, if the infection is refractory to treatment or is in the presence of spinal instability, surgery must be performed (Figure 2).

**Figure 3:**
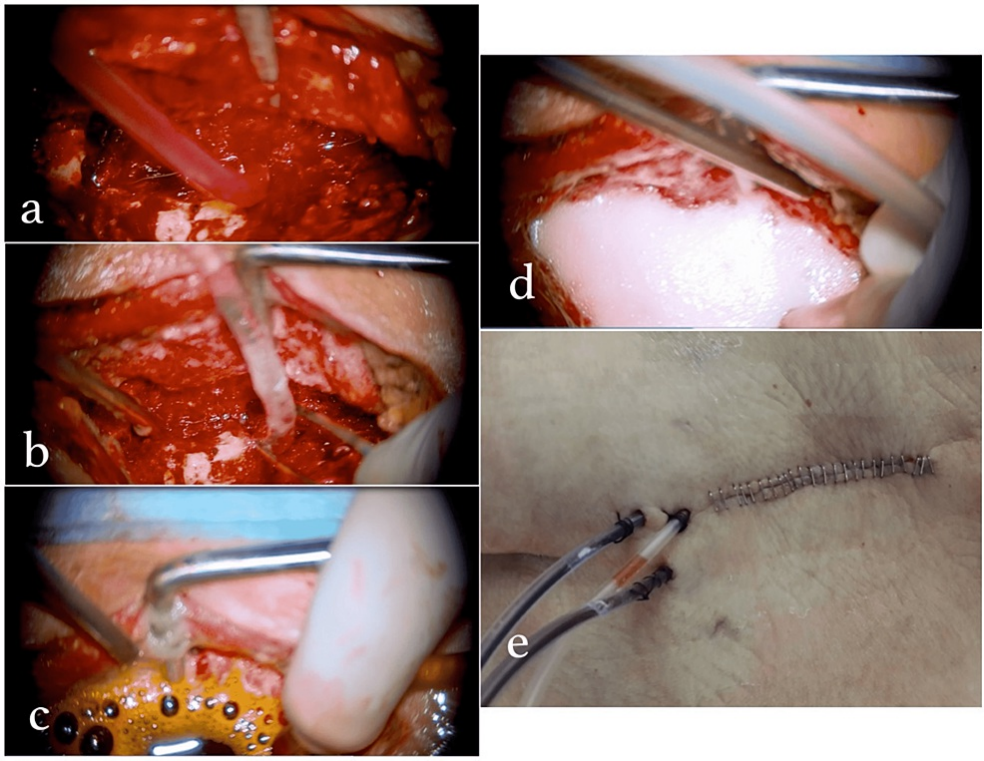
Images of the drainage system described in our algorithm a and b) Implanted silicone drainage catheter. c) Washing with batticon from drainage catheter. d) Washing with oxygenated water. e) The appearance of the drainage catheter on the skin surface together with hemovac drains

In surgery, we perform debridement-irrigation-drainage procedures, and in cases of instability, spinal instrumentation is performed. After surgery, we continue with irrigation and drainage procedures via a catheter three times a day (Figure 3).

Whether the patient had been operated on before, and if they were, the number of doctors involved in the operation, the amount of bleeding, and blood transfusion amounts were examined. WBC and ESR values, radiological findings, surgical method, culture results, and antibiotic regimens were reviewed at the time of diagnosis.

## Results

**Figure 4:**
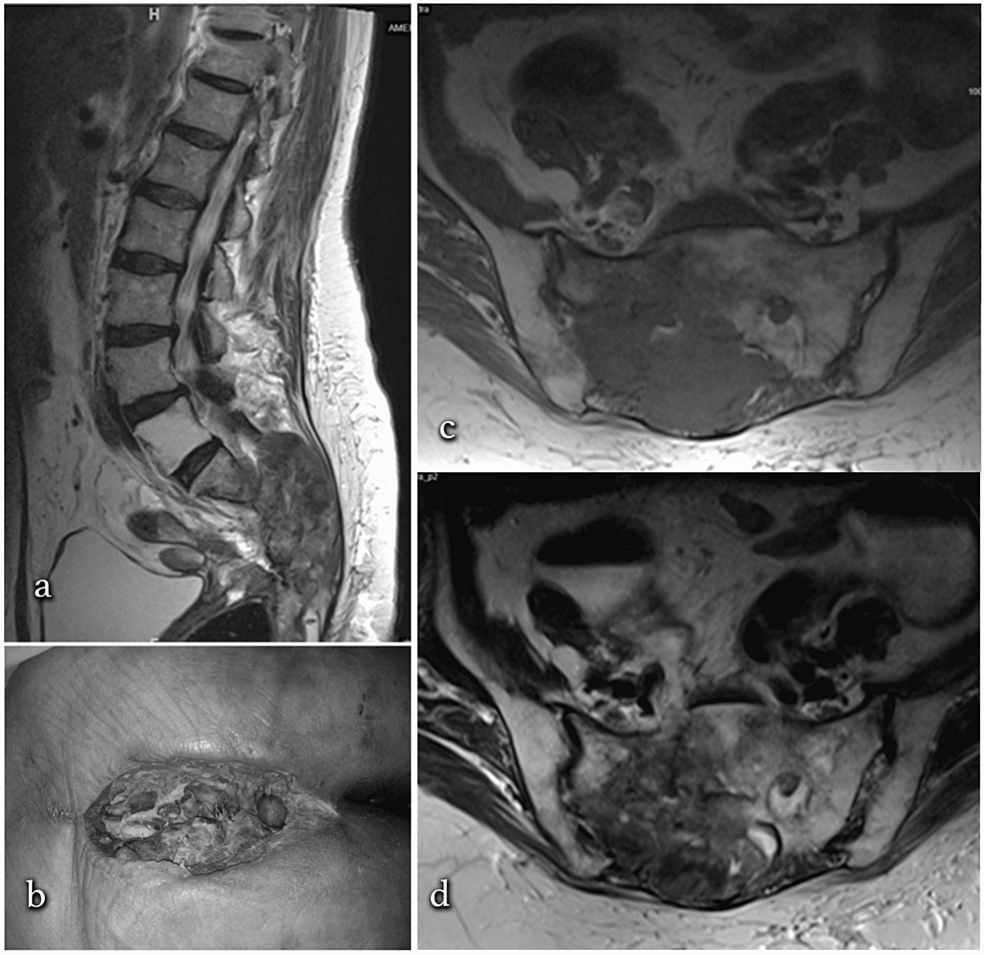
Abscess developing after sacral chordoma excision a) T2-weighted sagittal MRI image. b) Eventration of the skin surface in the infected area. c) T1-weighted axial MRI image. d) T2-weighted axial MRI image MRI: magnetic resonance imaging

**Figure 5:**
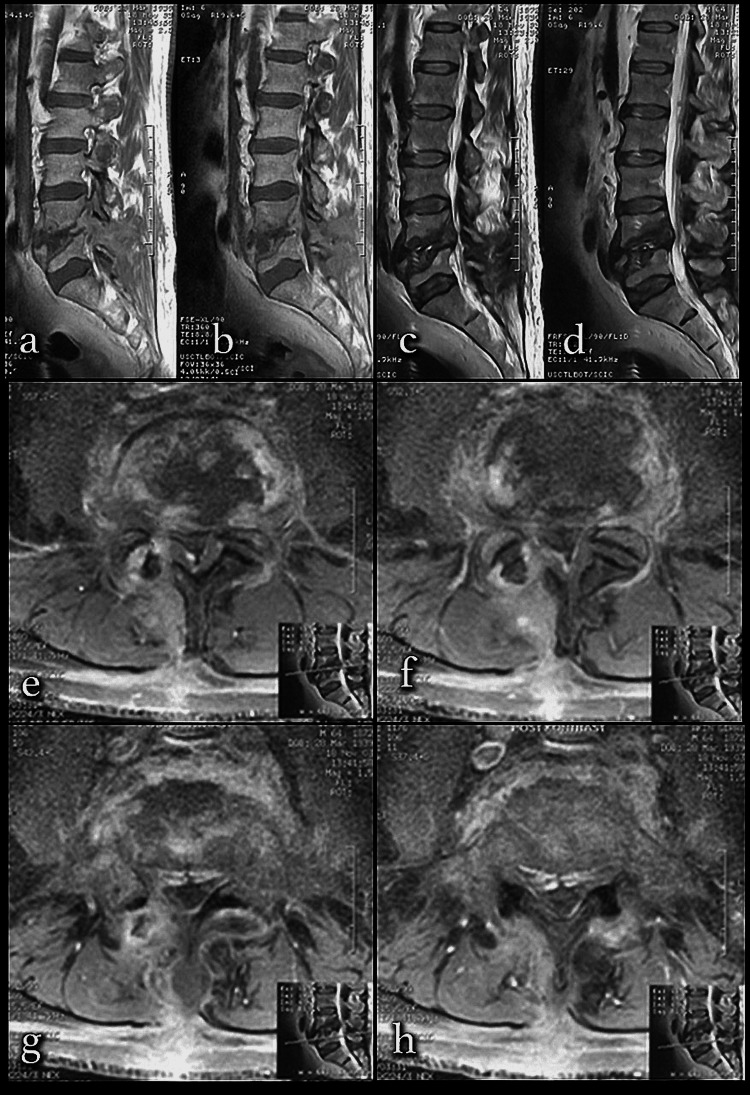
Case of L4-5 tuberculosis after microdiscectomy Destruction of the upper and lower endplates starting from the disk distance a, b, c, and d) T2-weighted sagittal MRI image. The infection, which started at the disc space, caused granuloma and destructed the lower and upper endplates. e and f) T2-weighted axial MRI images: left L4 hemilaminectomy area is seen. g and h) Contrast axial MRI images show destruction of the L4 endplate starting from disc space MRI: magnetic resonance imaging

Some examples of radiological and perioperative images pertaining to our cases are shown in Figures 4-5.

**Figure 6:**
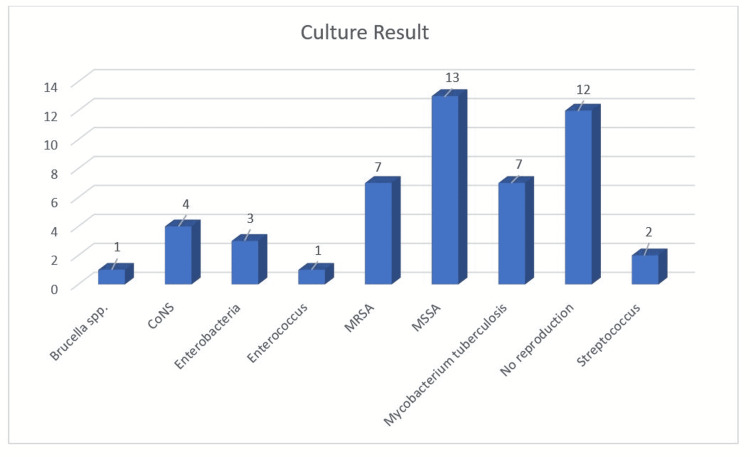
The percentages of detected microorganisms in culture results CoNS: coagulase-negative staphylococci; MRSA: methicillin-resistant *Staphylococcus aureus; *MSSA: methicillin-sensitive *Staphylococcus aureus*

Demographic information of 50 patients in our study, SI areas, previous surgeries if any, number of doctors participating in surgery and blood transfusion amounts, anatomical areas of SI, WBC at the time of diagnosis, ESR values, radiological findings at the time of diagnosis, interventional procedures for diagnosis and treatment, culture results, and antibiotic regimen are shown in Table 1, Table 2, and Figure 6. In 60% of patients, the area of infection was lumbar; it was thoracal in 4%, cervical in 12%, and sacral in 8%. The proportion of patients with multisegmental infection in the thoracolumbar region was 16%. Previously operated patients were diagnosed on the 30.16th day on average. The number of patients who were previously operated on in an external center and received SI treatment in our clinic was 18 (36%), and the number of patients diagnosed with SI after surgery in our clinic was 13 (26%). A total of 19 patients (38%) had no previous surgical history. The mean blood loss in previous surgeries of patients diagnosed with postoperative SI was 966 mL, the mean erythromycin suspension (ES) and fresh frozen plasma (FFP) transfusion amounts were 2,071 Ü ES and 1.5 Ü FFP, and the average number of physicians participating in surgery was 2.64. The average WBC count of the patients at the time of diagnosis was 13.48 K/uL, and the average ESR value was 41.66 mm. Radiologically, the most common finding was spondylodiscitis/discitis (32%). Osteomyelitis was detected in one (2%) patient. Methicillin-sensitive *Staphylococcus aureus *(MSSA) was the most commonly isolated organism in culture results and was detected in 13 patients (26%). Culture results in 12 patients (24%) were negative (Figure 6).

The number of patients with active SIs who were unstable and stabilized at the time of diagnosis was 11 (22%) and the existing stabilization materials of two patients (4%) were removed. The overall findings are presented in Table 1 and Table 2.

**Table 1:** The infection parameters, diagnosis, culture results, and treatment modalities of all patients CHNP: cervical herniated nucleus pulposus; LDH: lumbar disc herniation; LHNP: lumbar herniated nucleus pulposus; ACD: anterior cervical discectomy; CSF: cerebrospinal fluid; ES: erythromycin suspension; FFP: fresh frozen plasma

Case no.	Prior diagnosis	Age, years	Sex	Region	Prior operation	Prior operation drainage	Prior operation EBL cc.	Prior operation transfusion	No. of surgeons
1	Spinal infection, TBC?	39	M	Thoracic	None	None	None	None	None
2	Spontaneouseous epidural abscess	67	M	Lumbar	None	None	None	None	None
3	Spondylolisthesis + stenosis	85	M	Lumbar	Pedicle screw, L3-5, dynamic system	D+	1,200	2 Ü ES+ 2 Ü FFP	3
4	Degenerative spondylosis + canal stenosis	41	M	Thoracolumbar	Pedicle screw, T10-S1	D+	950	1 Ü ES+ 1 Ü FFP	3
5	Spinal infection, TBC?	62	F	Lumbar	None	None	None	None	None
6	Spondylolisthesis + stenosis	70	M	Sacral	Pedicle screw, L5-S1	D+	500	1 Ü ES	2
7	Degenerative spondylosis + canal stenosis	53	M	Thoracolumbar	Pedicle screw, L3-S1, dynamic system	D+	750	1Ü ES+ 1Ü FFP	2
8	Spontaneous epidural abscess	47	M	Lumbar	None	None	none	None	None
9	Cervical myelopathy	73	M	Cervical	Screw C3-6	D+	7,500	1 Ü ES+ 1 Ü FFP	3
10	CHNP	65	M	Cervical	ACD, C5-6	D-	100	None	2
11	Spinal infection, TBC?	40	F	Lumbar	None	None	None	None	None
12	Spontaneous epidural abscess	72	F	Lumbar	None	None	None	None	None
13	Spinal infection, TBC?	69	F	Lumbar	None	None	None	None	None
14	Spontaneous epidural abscess	37	M	Lumbar	None	None	None	None	None
15	LDH	53	M	Lumbar	LDH, L4-5	D-	150	None	2
16	L1 + L2 fracture	79	M	Thoracolumbar	Pedicle screw, T10-iliac wing, dynamic system	D+	1,800	3 Ü ES + 1 Ü FFP	3
17	Spondylolisthesis + stenosis	61	M	Thoracolumbar	Pedicle screw L3-S1	D+	450	None	3
18	Spinal infection, TBC?	67	F	Lumbar	None	None	None	None	None
19	Spinal infection, TBC?	70	M	Thoracic	None	None	None	None	None
20	Degenerative spondylosis + canal stenosis	87	F	Sacral	Pedicle screw, L3-S1, dynamic system	D+	450	None	2
21	Degenerative spondylosis + canal stenosis	47	M	Thoracolumbar	Pedicle screw, T10-iliac wing, dynamic system	D+	2,200	3 Ü ES+ 1 Ü FFP	3
22	CHNP	61	F	Cervical	ACD, C5-6	D-	200	None	2
23	LHNP	74	F	Lumbar	LDH, L5-S1	D-	150	None	2
24	T11 + T12 Fracture	57	M	Thoracolumbar	Pedicle screw, T10-L1+ T11-T12 kyphoplasty, dynamic system	D+	1,300	2 Ü ES+ 1 Ü FFP	3
25	Spondylolysis	53	M	Lumbar	None	None	None	None	None
26	Spinal infection, pyogenic?	69	M	Lumbar	None	None	None	None	None
27	CHNP	47	M	Cervical	ACD, C5-6	D-	100	None	2
28	Spontaneous epidural abscess	77	M	Lumbar	None	None	None	None	None
29	Spinal stenosis	67	M	Lumbar	Pedicle screw, L3-5	D+	450	None	2
30	LHNP	39	F	Lumbar	LDH, L4-5; CSF fistula repair	D+	150	None	2
31	Spinal infection, TBC?	58	F	Lumbar	None	None	None	None	None
32	Spondylolisthesis + stenosis	82	F	Lumbar	Pedicle screw, L3-S1, dynamic system	D+	750	1 Ü ES+ 1 Ü FFP	2
33	Spontaneous epidural abscess	64	M	Lumbar	None	None	None	None	None
34	Spontaneous epidural abscess	73	M	Lumbar	None	None	None	None	None
35	Spontaneous epidural abscess	69	F	Lumbar	None	None	None	None	None
36	Sacrum tumor	81	F	Sacral	Sacral tumor excision	D+	3,000	4 Ü ES + 3 Ü FFP	4
37	Spinal infection, brucellosis?	51	F	Lumbar	None	None	None	None	None
38	Spontaneous epidural abscess	58	M	Lumbar	None	None	None	None	None
39	CHNP	76	F	Cervical	ACD, C5-6+ C6-7	D-	100	None	2
40	CHNP	59	M	Cervical	ACD, mini plac+ screw	D+	100	None	3
41	LHNP	50	F	Lumbar	LDH, L4-5	None	100	None	2
42	LHNP	72	M	Lumbar	LDH, L5-S1	None	100	None	2
43	Degenerative spondylosis + canal stenosis	62	F	Thoracolumbar	Pedicle screw, T10-iliac wing, dynamic system	D+	1,800	3 Ü ES + 2 Ü FFP	3
44	LHNP	75	F	Lumbar	LDH, L3-4	D-	100	None	2
45	LHNP	63	F	Lumbar	LDH, L4-5	D-	150	None	2
46	Sacrum tumor	73	M	Sacral	Sacral tumor excision	D+	2,500	3 Ü ES+ 3 Ü FFP	3
47	LHNP	69	M	Lumbar	LDH, L4-5	D-	100	None	2
48	L1 fracture	53	F	Thoracolumbar	Pedicle screw, T10-L3	D+	1,100	2 Ü ES	3
49	Spondylolisthesis + stenosis	67	F	Lumbar	Pedicle screw L4-5	D+	350	None	3
50	Degenerative spondylosis + canal stenosis	73	F	Lumbar	Pedicle screw, T12-iliac wing, dynamic system	D+	1,300	2 Ü ES+ 1 Ü FFP	3

**Table 2:** Demographic features, prior diagnosis, prior surgery, prior transfusion amount, and number of surgeons participating in prior surgery MSSA: Methicillin-sensitive *Staphylococcus aureus; *MRSA: methicillin-resistant Staphylococcus aureus; CoNS: coagulase-negative staphylococci

Case no.	Postop. day	WBC, K/uL	ESR, mm.	Radiological finding	Surgery for infection	Culture result	Antibiotic therapy
1	\	11.3	37	Paradiscal involvement	Debridement + irrigation	Mycobacterium TBC	IZH 3 x 100 mg + RIF 2 x 300 mg + PZA 4 x 400 mg 2 months, IZH 3 x 100 mg + RIF 2 x 300 mg + EMB 1 x 400 mg 3 times a week for 4 months
2	\	14.7	57	Spinal stenosis	Debridement + irrigation + stabilization + vancomycin powder 1 gr	MSSA	SAM 4 x 1 gr, GEN 2 x 80 mgr
3	8	19.4	74	Endplate destruction	Debridement + irrigation + vancomycin powder 1 gr	MRSA	VAN 2 x 1 gr, GEN 1 x 160 mgr (10 days), after TMP-SMX 4 weeks oral, 8 weeks
4	21	17.1	63	Paraspinal abscess	Debridement + irrigation + vancomycin powder 1 gr	MRSA	VAN 2 x 1 gr, GEN 1 x 160 mgr (10 days), after TMP-SMX 4 weeks oral, 8 weeks
5	\	11.7	37	Disc space sparing	Debridement + irrigation	Mycobacterium TBC	IZH 3 x 100 mg + RIF 2 x 300 mg + PZA 4 x 400 mg 2 months, IZH 3 x 100 mg + RIF 2 x 300 mg + EMB 1 x 400 mg 3 times a week for 4 months
6	12	7.8	25	Inflammation and small abscess	Debridement + irrigation + vancomycin powder 1 gr	No reproduction	CFZ 2 x 1 gr, 4 weeks
7	700	8.4	27	High bone destruction	Debridement + irrigation + stabilization material removal + vancomycin	MSSA	SAM 4 x 1 gr, GEN 2 x 80 mgr, 6 weeks
8	\	12.6	46	Spinal canal involvement	Debridement + irrigation + stabilization + vancomycin powder 1 gr	CoNS	CIP 2 x 200 mgr, AMC 3 x 625 mgr, 5 weeks
9	28	14.5	52	Spondylodiscitis	Debridement irrigation + vancomycin powder 1 gr	MSSA	VAN 2 x 1gr, GEN 1 x 160 mgr (10 days), after TMP-SMX 4 weeks oral, 8 weeks
10	4	13.2	51	Discitis	Debridement irrigation	Enterobacteria	MEM 2 x 1 gr, CST 2 x 150 mg 2 weeks
11	\	5.4	19	Subligamentous involvement	Debridement + irrigation	Mycobacterium TBC	IZH 3 x 100 mg + RIF 2 x 300 mg + PZA 4 x 400 mg 2 months, IZH 3 x 100 mg + RIF 2 x 300 mg + EMB 1 x 400 mg 3 times a week for 4 months
12	\	6.8	23	Spinal canal involvement	Debridement + irrigation + stabilization + vancomycin powder 1 gr	CoNS	CIP 2 x 200 mgr, AMC 3 x 625 mgr, 5 weeks
13	\	15.1	60	Endplate destruction	Debridement + irrigation	Mycobacterium TBC	IZH 3 x 100 mg + RIF 2 x 300 mg + PZA 4 x 400 mg 2 months, IZH 3 x 100 mg + RIF 2 x 300 mg + EMB 1 x 400 mg 3 times a week for 4 months
14	\	9.7	32	Spinal canal involvement	Debridement + irrigation +stabilization + vancomycin powder 1gr	No reproduction	Postoperative 4 days VAN 4 x 500 mgr, AMK 1 x 500 mgr, after discharge SAM 2 x 37, 1 week I.V and 4 weeks oral
15	21	16.2	59	Spondylodiscitis	Debridement + irrigation + vancomycin powder 1 gr	No reproduction	CTX 2 x 1 gr, 4 weeks
16	15	14.0	53	Skip small abscess	Debridement + irrigation + vancomycin powder 1 gr	MSSA	SAM 4 x 1 gr, GEN 2 x 80 mgr, 6 weeks
17	34	7.8	23	Paraspinal abscess	Debridement + irrigation + vancomycin powder 1 gr	No reproduction	CFZ 2 x 1 gr, 4 weeks
18	\	11.9	35	High bone destruction	Debridement + irrigation	Mycobacterium TBC	IZH 3 x 100 mg + RIF 2 x 300 mg + PZA 4 x 400 mg 2 months, IZH 3 x 100 mg + RIF 2 x 300 mg + EMB 1 x 400 mg 3 times a week for 4 months
19	\	10.8	33	Endplate destruction	Debridement + irrigation	Mycobacterium TBC	IZH 3 x 100 mg + RIF 2 x 300 mg + PZA 4 x 400 mg 2 months, IZH 3 x 100 mg + RIF 2 x 300 mg + EMB 1 x 400 mg 3 times a week for 4 months
20	11	8.7	29	Subligamentous abscess	Debridement + irrigation + vancomycin powder 1 gr	Enterococcus	VAN 2 x 1 gr 2 weeks
21	19	16.7	61	Discitis	Debridement + irrigation + vancomycin powder 1 gr	MRSA	GEN 10 days 1 x 160 mgr, 6 weeks VAN 10 days 2 x 1 gr, after discharge SAM 2 x 375 mgr, 6 weeks I.V. and 4 weeks oral
22	7	19.0	77	Spondylodiscitis	Debridement + irrigation + vancomycin powder 1 gr	MRSA	VAN 2 x 1 gr, GEN 1 x 160 mgr (10 days), after TMP-SMX 4 weeks oral, 8 weeks
23	33	12.1	40	Spondylodiscitis	Debridement + irrigation + vancomycin powder 1 gr	MSSA	SAM 4 x 1 gr, GEN 2 x 80 mgr, 6 weeks
24	28	13.6	44	Osteomyelitis	Debridement + irrigation + vancomycin powder 1 gr	CoNS	CIP 2 x 200 mgr, AMC 3 x 625 mgr, 5 weeks
25	\	8.3	27	Facet joint arthritis	Debridement + irrigation + stabilization + vancomycin powder 1 gr	No reproduction	CFZ 2 x 1 gr, 4 weeks
26	\	10.4	36	Subdural involvement	Debridement + irrigation + stabilization + vancomycin powder 1 gr	Streptococcus	AMC 3 x 625 mgr, 4 weeks
27	12	16.4	64	Discitis	Debridement + irrigation + vancomycin powder 1 gr	MRSA	GEN 10 days 1 x 160 mgr, 6 weeks VAN 10 days 2 x 1 gr, after discharge SAM 2 x 375 mgr, 6 weeks I.V. and 4 weeks oral
28	\	11.3	33	Spinal canal involvement	Debridement + irrigation + stabilization + vancomycin powder 1 gr	No reproduction	Postoperative 4-day VAN 4 x 500 mgr, AMK 1 x 500 mgr, after discharge SAM 2 x 375 mg, 1-week I.V. and 4 weeks oral
29	70	6.7	24	Spinal canal stenosis	Debridement + irrigation + vancomycin powder 1 gr	No reproduction	CFZ 2 x 1 gr, 4 weeks
30	15	12.7	42	Subdural empyema	Debridement + irrigation + vancomycin powder 1 gr	Enterobacteria	MEM 2 x 1 gr + CST 2 x 150 mg 2 weeks
31	\	8.3	28	Disc space sparing	Debridement + irrigation	Mycobacterium TBC	IZH 3 x 100 mg + RIF 2 x 300 mg + PZA 4 x 400 mg 2 months, IZH 3 x 100 mg + RIF 2 x 300 mg + EMB 1 x 400 mg 3 times a week for 4 months
32	42	12.0	39	Spondylodiscitis	Debridement + irrigation + vancomycin powder 1 gr	CoNS	CIP 2 x 200 mgr, AMC 3 x 625 mgr, 5 weeks
33	\	11.2	30	Spinal canal involvement	Debridement + irrigation + stabilization + vancomycin powder 1 gr	MSSA	SAM 4 x 1 gr, GEN 2 x 80 mgr, 6 weeks
34	\	7.4	25	Spinal canal involvement	Debridement + irrigation + stabilization + vancomycin powder 1 gr	No reproduction	CFZ 2 x 1 gr, 4 weeks
35	\	5.8	19	Spinal canal involvement	Debridement + irrigation + stabilization + vancomycin powder 1 gr	No reproduction	Postoperative 4-day VAN 4 x 500 mgr, AMK 1 x 500 mgr, after discharge SAM 2 x 375 mg, 1-week I.V. and 4 weeks oral
36	28	13.6	47	Spondylodiscitis	Debridement + irrigation + vancomycin powder 1 gr	MSSA	SAM 4 x 1 gr, GEN 2 x 80 mgr, 6 weeks
37	\	12.9	44	Spondylodiscitis	Debridement + irrigation + vancomycin powder 1 gr	Brucella spp.	Doxycycline 2 x 100 mg (6 weeks) + RIF 3 x 300 mg (3 weeks)
38	\	10.3	31	Spinal canal involvement	Debridement + irrigation + stabilization + vancomycin powder 1 gr	MSSA	SAM 4 x 1 gr, GEN 2 x 80 mgr, 6 weeks
39	4	16.7	58	Spondylodiscitis	Debridement + irrigation + vancomycin powder 1 gr	MRSA	GEN 10 days 1 x 160 mgr, 6 weeks VAN 10 days 2 x 1 gr, after discharge SAM 2 x 375 mgr, 6 weeks I.V. and 4 weeks oral
40	7	22.4	84	Spondylodiscitis	Debridement + irrigation + vancomycin powder 1 gr	MSSA	SAM 4 x 1 gr, GEN 2 x 80 mgr, 6 weeks
41	21	13.6	49	Inflammation and small abscess	Debridement + irrigation + vancomycin powder 1 gr	Enterobacteria	MEM 2 x 1 gr + CST 2 x 150 mg, 4 weeks
42	14	11.8	37	Inflammation and paraspinal abscess	Debridement + irrigation + vancomycin powder 1 gr	MSSA	SAM 4 x 1 gr, GEN 2 x 80 mgr, 6 weeks
43	45	8.7	31	Paraspinal abscess	Debridement + irrigation + vancomycin powder 1 gr	MSSA	SAM 4 x 1 gr, GEN 2 x 80 mgr, 6 weeks
44	28	11.4	34	Discitis	Debridement + irrigation + vancomycin powder 1 gr	Streptococcus	AMC 3 x 625 mgr, 4 weeks
45	14	13.0	44	Discitis	Debridement + irrigation + vancomycin powder 1 gr	No reproduction	CFZ 2 x 1 gr, 4 weeks
46	52	7.1	23	Paraspinal abscess and hematoma	Debridement + irrigation + vancomycin powder 1 gr	MSSA	SAM 4 x 1 gr, GEN 2 x 80 mgr, 6 weeks
47	7	17.8	72	Discitis	Debridement + irrigation + vancomycin powder 1 gr	MRSA	VAN 2 x 1 gr, GEN 1 x 160 mgr (10 days), after TMP-SMX 4 weeks oral, 8 weeks
48	72	8.6	29	Paraspinal abscess	Debridement + irrigation + vancomycin powder 1 gr	No reproduction	Postoperative 4-day VAN 4 x 500 mgr, AMK 1 x 500 mgr, after discharge SAM 2 x 375 mg, 1-week I.V. and 4 weeks oral
49	60	9.4	34	Paraspinal abscess	Debridement + irrigation + vancomycin powder 1 gr	MSSA	SAM 4 x 1 gr, GEN 2 x 80 mgr, 6 weeks
50	133	11.9	42	Spondylodiscitis	Debridement + irrigation + stabilization material removal + vancomycin	No reproduction	CFZ 2 x 1 gr, 4 weeks

## Discussion

Diagnostic and treatment strategies for SI are still controversial. There are reports of individual diagnostic and treatment algorithms, and clinics follow a modified approach according to their own experiences. In our study, we describe the most effective diagnostic and treatment options by offering a vigorous diagnostic-treatment algorithm for widespread use.

There are numerous studies on surgical treatment with debridement-irrigation [25]; however, they are limited to infections occurring within 30-90 days after surgery [25]. We found intermittent irrigation (three times a day) with oxygen water and batticon through foley catheter sweat in the surgical treatment of SI patients to be effective in light of our own experience. We also determined the duration of the intermediate irrigation application with the decrease in the patient's CRP values. When the CRP value decreased to half the initial value, we stopped irrigation and removed the catheter. We also used stabilization and debridement plus intermittent irrigation in unstable patients or in patients diagnosed before the fourth month of treatment. Many reports advise the removal of existing stabilization materials when SI is detected [2]. Also, most reports recommend stabilization after SI has been treated [26]. At our own clinic, we recommend stabilizing patients with SI with unstable vertebrae before four months, even if there is an early SI, and treating them with debridement plus intermittent irrigation. This ensures that the patient is mobilized in the early period, as well as assuring the possibility of treating his or her primary pathology with a single intervention. As we indicated in our algorithm, intravenous antibiotic treatment is also started in accordance with the antibiotic result for all our patients and culture-negative patients who prefer broad-spectrum antibiotics. Follow-up is carried out with ESR and CRP values in accordance with the literature, and we also consider the value of procalcitonin in treatment-resistant patients.

In our study, all patients were treated successfully by the 180th day after SI was detected. This value of 180 days also applies to patients with tuberculosis whose treatment lasted longer. One patient developed osteomyelitis and the existing stabilization material of two patients was removed because the material became infected. Conversely, patients with SI and unstable spine for more than four months after the first detection were included in the group of patients with resistant spinal infections. Early stabilization was not preferred for these patients, and the SI was stabilized after treatment. Stabilization materials, if used in these patients, were removed. In our study, one patient (2%) was treated in this manner. There were 19 patients (38%) in our study who were first diagnosed with SI at our clinic. Seven of these patients were diagnosed with tuberculosis (14%) (Figure 5), and one was diagnosed with brucellosis (2%). A total of 12 patients (24%) were stabilized early, and the form of stabilization was rigid, semi-rigid, or dynamic in accordance with the patient’s pathology. In addition to debridement and intermittent irrigation, vancomycin powder (1 g) was sprinkled in the surgical lodgings. It has been reported that this procedure has an effect on Gram-positive microorganisms [27], although some researchers have suggested that this practice may lead to an increase in vancomycin-resistant bacteria or an increase in Gram-negative bacterial-heavy infection [28].

Because standard treatment of tuberculosis disease can extend to six months, our study was based on a six-month period. All our patients with SI were treated based on clinical, laboratory, and radiological parameters. Only two patients (4%) needed to have the stabilization material removed.

In 2012, Gasbarrini et al. published a comparative study in which needle biopsy accompanied by percutaneous CT was used to diagnose unknown spinal lesions, and its accuracy rate was reported to be as high as 70%. However, the diagnostic efficiency of CT-accompanied needle biopsy may vary among centers depending on the expertise of the radiologist, the number of samples sent, and whether the patient had undergone previous antibiotic therapy. Therefore, some researchers recommend clear biopsies for patients with negative CT-accompanied cultures. Other investigators report that a biopsy accompanied by a second CT may be beneficial [29]. Based on the experiences at our clinic, in patients with an irrefutable surgery indication, open biopsy is the first choice, because the biopsy method allows for the collection of a large amount of tissue. For patients who do not meet surgical criteria and for whom a histological diagnosis is critical, a CT-accompanied biopsy is the first option.

Today, the diagnosis of radionuclide should be preferred for patients with an indefinite diagnosis or who require individualized follow-up [23]. In our algorithm, it is recommended that scintigraphy be performed.

In 24% of our patients, no pathogens were reproduced or produced as a result of culture results. These patients were treated in accordance with our algorithm and broad-spectrum antibiotic treatment was initiated. MSSA was the most common organism detected in cultural results (26%), followed by MRSA (14%), *M. tuberculosis* (14%), coagulase-negative staphylococci (CoNS) (8%), Enterobacteria (6%), Streptococcus (4%), and Enterococcus (2%) (Figure 6). Polymicrobial reproduction was not detected in any of our patients.

In our study, it was determined that more than two surgeons had participated in the operation and that the rate of SI was high enough in patients with perioperative bleeding to require erythromycin suspension and fresh frozen plasma. These findings are compatible with the existing literature [30].

There are several limitations to our study. Firstly, the initial CRP value for all patients was not considered in the diagnosis of SI. The reason is that CRP values were not obtained for the patients initially diagnosed and treated at an external center at the time of admission. Therefore, this important parameter is not considered in our analysis together with WBC and ESR. However, CRP value was included during the treatment process of the patients at our clinic and used as a parameter in the evaluation of the effectiveness of the treatment. The second limitation is that patients with tuberculosis were also included in our study. Some critics may argue that including these patients would affect the homogeneity of the study. However, we also wanted to share these data, especially when we determined that approximately 14% of SI detected for first-time occurred in patients with tuberculosis. We also used our standard algorithm in patients with SI with tuberculosis. The third limitation is that including negative anaerobic culture results could have made the study more comprehensive. Another limitation is that the study was retrospective in design. We strongly recommend a prospective study that avoids these limitations.

Clinical implications

Due to the diverse patient population and treatment options, there is no generally applicable diagnostic-treatment guideline for SI. We recommend early surgery, especially in elderly and critical patients, because many patients present in the preseptic phase and often deteriorate during treatment. At the early stage of infection, less invasive procedures may still be preferred, whereas, in later stages, more serious conditions are commonly caused by large bone loss and deterioration of the sagittal balance of the spine and may require extensive resections and long-segment stabilization. In severely affected patients with wide, deep infection, and extremely high levels of CRP and ESR, we recommend surgery if medically appropriate. However, further prospective randomized trials are needed to validate our diagnosis and treatment strategies.

## Conclusions

SI is a very challenging condition in terms of both the treatment process and patient satisfaction. In order for patients to return to their normal everyday lives as soon as possible, it is critical to make a prompt diagnosis and to choose the appropriate treatment option. In this study, we shared our own experiences on this subject and offered a diagnostic-treatment algorithm that can help with SI management.
